# Combination of transcriptional biomarkers and clinical parameters for early prediction of sepsis indued acute respiratory distress syndrome

**DOI:** 10.3389/fimmu.2022.1084568

**Published:** 2023-01-04

**Authors:** Ren-Qi Yao, Zong Shen, Qi-Min Ma, Ping Ling, Chen-Ru Wei, Li-Yu Zheng, Yu Duan, Wei Li, Feng Zhu, Yu Sun, Guo-Sheng Wu

**Affiliations:** ^1^ Department of Burn Surgery, The First Affiliated Hospital of Naval Medical University, Shanghai, China; ^2^ Translational Medicine Research Center, Medical Innovation Research Division and Fourth Medical Center of the Chinese People’s Liberation Army (PLA) General Hospital, Beijing, China

**Keywords:** sepsis, acute respiratory distress syndrome, prediction, biomarker, gene expression

## Abstract

**Objective:**

As a common yet intractable complication of severe sepsis, acute respiratory distress syndrome (ARDS) is closely associated with poor clinical outcomes and elevated medical expenses. The aim of the current study is to generate a model combining transcriptional biomarkers and clinical parameters to alarm the development of ARDS in septic patients.

**Methods:**

Gene expression profile (GSE66890) was downloaded from the Gene Expression Omnibus database and clinical data were extracted. Differentially expressed genes (DEGs) from whole blood leukocytes were identified between patients with sepsis alone and septic patients who develop ARDS. ARDS prediction model was constructed using backward stepwise regression and Akaike Information Criterion (AIC). Meanwhile, a nomogram based on this model was established, with subsequent internal validation.

**Results:**

A total of 57 severe septic patients were enrolled in this study, and 28 (49.1%) developed ARDS. Based on the differential expression analysis, six DEGs (BPI, OLFM4, LCN2, CD24, MMP8 and MME) were screened. According to the outcome prediction model, six valuable risk factors (direct lung injury, shock, tumor, BPI, MME and MMP8) were incorporated into a nomogram, which was used to predict the onset of ARDS in septic patients. The calibration curves of the nomogram showed good consistency between the probabilities and observed values. The decision curve analysis also revealed the potential clinical usefulness of the nomogram. The area under the receiver operating characteristic (AUROC) for the prediction of ARDS occurrence in septic patients by the nomogram was 0.86 (95% CI = 0.767-0.952). A sensitivity analysis showed that the AUROC for the prediction of ARDS development in septic patients without direct lung injury was 0.967 (95% CI = 0.896-1.0).

**Conclusions:**

The nomogram based on transcriptional biomarkers and clinical parameters showed a good performance for the prediction of ARDS occurrence in septic patients.

## Introduction

Acute respiratory distress syndrome (ARDS), a severe form of lung injury, represents a common consequence of pneumonia, trauma, shock, inhalation injury, acute pancreatitis and high-risk surgery. Meanwhile, ARDS also remains a devastating complication of severe sepsis, rendering septic patients at greater risk of in-hospital death (1). It is estimated that there are more than 210,000 cases of sepsis-induced ARDS (Se-ARDS) annually in the United States. Notably, patients with Se-ARDS have higher case fatality rates than those with ARDS caused by other reasons (2). Therefore, early prediction, recognition, and identification of Se-ARDS could prompt offering of optimal and goal-directed therapy.

Numerous studies have examined the role of biomarkers in ARDS from multiple dimensions, including diagnosis, prognosis and prediction (3). It has been widely accepted that a panel of biomarkers was superior to individual clinical parameter or biomarker in predicting or diagnosing ARDS (4–6). However, majority of earlier studies merely focused on predictive value of clinical biochemical indexes, serum level of inflammatory mediators, as well as protein molecules correlating with endothelial and epithelial injury (3). In addition, ARDS is a highly heterogeneous syndrome, and biomarker levels have been shown to substantially differ across ARDS resulted from disparate causes (7). Seldom studies have explored the potential significance of a panel of biomarkers in predicting the development of ARDS in patients with severe sepsis (6).

To date, transcriptomic-based research bring about encouraging results in the field of infectious diseases, including sepsis/Se-ARDS (8, 9). Bioinformatic studies especially on the changes of gene expression network have revealed the differences in gene expression between patients with sepsis alone and septic patients complicated with ARDS (9–11). In the present study, we screened the transcriptional indicators for the occurrence of ARDS among septic patients using systemic and comprehensive bioinformatics methods, followed by establishment of a predictive model containing a small panel of transcriptional and clinical biomarkers, thereby prompting the identification of septic patients at greater risk for developing ARDS.

## Materials and methods

### Data source

This research included data of septic patients from a previously published prospective study, which enrolled critically ill patients admitted to a tertiary care hospital intensive care unit (ICU) (12). The original trial was approved by the Institutional Review Board of the University of California, San Francisco. Informed consent was obtained as described in another previous paper. Raw data (GSE66890) were available in the Gene Expression Omnibus (GEO) database (https://www.ncbi.nlm.nih.gov/geo/). The secondary analyses of the data were approved by the ethics committee of the Changhai hospital.

### Study population and data extraction

The patients were selected according to the inclusion and exclusion criteria adopted by the original study. The details of the criteria have been described clearly, in which sepsis was defined as documented or suspected infection along with presence of two or more characteristics of systemic inflammatory response syndrome (SIRS) (12). Following demographic and laboratory data were extracted: age, gender, etiology of lung injury, Acute Physiology and Chronic Health Evaluation III (APACHE III), tumor, presence of shock, absolute neutrophil count (ANC), white blood cell count (WBC), and serum creatinine. All the data were collected within 24 h of admission to the ICU.

### Outcome measurement

The primary outcome of this study represented the development of ARDS. In the present study, the Berlin definition of ARDS was applied. The secondary endpoint represents 60-day mortality, which was defined as in-hospital death up to day 60 of follow-up.

### Microarray data

The original expression profile data of GSE66890 was downloaded from the GEO database. Twenty-nine whole blood samples collected from patients with sepsis alone and twenty-eight samples from septic patients with ARDS were included in GSE66890. Gene expression profiles were generated using Affymetrix Human GeneChip Gene 1.0 ST array (Affymetrix, Santa Clara, CA). Detailed methodologies on RNA extraction and microarray hybridization can be found in the GEO database.

### Identification of the differentially expressed genes

Differentially expressed genes (DEGs) screening was performed between the patients with sepsis alone and septic patients with ARDS using ‘Limma’ package. A *p* value < 0.05 and |log fold change (FC)| > 1 were considered as of statistical significance. The DEGs were visualized using a volcano plot and a heatmap with ‘ggplot2’ and ‘pheatmap’ package, respectively. Upregulated and downregulated DEGs were identified independently.

### Construction of gene network and functional annotation

To further explore latent biological function of these genes, a gene network was constructed based on the Genemania database (http://genemania.org/). The analysis parameters are as follows: max resultant genes=20; max resultant attributes=10. Functional annotation was carried out using Gene Ontology (GO) items.

### Statistical analysis

Continuous data in normal distribution were expressed as mean ± standard deviation, whereas non-normally distributed continuous variables were summarized as median (interquartile range). Categorical variables were presented as numbers or percentage. Student’s *t* test, One-way analysis of variance (ANOVA), Mann-Whitney *U* test, Chi-square test or Fisher’s exact test were used as appropriate. Univariable logistic regression analyses were performed and the statistically significant variables with *p* value less than 0.05 were further selected for multivariable logistic regression analyses. According to the Akaike Information Criterion (AIC), the prediction model corresponding to the minimum AIC value was selected. The calibration plot was adopted to assess the goodness of fit of the model, and decision curve analysis (DCA) was applied to evaluate the benefits. The concordance index (c-index) was used to determine the discriminatory capacity of the model. The Hosmer–Lemeshow goodness of fit test was conducted to validate the calibration. The receiver operating characteristic (ROC) curve was used to calculate the optimal diagnostic cut-off value. All statistical analyses were carried out using R software (version 4.1.3) and SPSS software (version 21.0). A two-tailed *p* value less than 0.05 was deemed as of statistical significance.

## Results

### Baseline characteristics of enrolled patients

The clinical characteristics of the included patients from GSE66890 dataset was shown in **Table 1**. The mean age of this cohort was 62.74 ± 20.01 years, of which 32 (56.1%) were males. Among a total of 57 septic patients, 28 (49.1%) developed ARDS. Of note, direct lung injury as an etiology of ARDS was more prevalent in septic patients with ARDS (72.4%) than those without ARDS (46.4%). ARDS was apt to occur in septic patients with shock as well as those with higher APACHE III scores. Conversely, ARDS was rarely observed in patients without tumor. There was no difference in age, gender, ANC, WBC and creatinine between septic patients with or without ARDS. The incidence of end-stage renal disease (ESRD) and death at 60 days were 10.3% and 31% in septic patients with ARDS and 17.9% and 17.9% in septic patients without ARDS, respectively.

**Table 1:** Baseline characteristics, laboratory parameters and clinical outcomes stratified by the ARDS occurrence.

Characteristics	Total	With ARDS	No ARDS	*p* value
Age	62.74 ± 20.01	58.62 ± 19.12	67.0 ± 20.32	0.882
Male	32	16 (55.2%)	16 (57.1)	0.881
Direct lung injury^*^	34	21 (72.4%)	13 (46.4%)	0.046
Shock	33	21 (72.4%)	12 (42.9%)	0.024
APACHE III	103.58 ± 39.43	119.76 ± 29.88	86.82 ± 31.66	0.041
ANC	11.28 ± 6.95	9.82 ± 7.09	12.8 ± 6.59	0.897
WBC	12.78 ± 7.17	10.81 ± 6.97	14.81 ± 6.91	0.733
Creatinine	1.37 (0.87, 2.5)	1.45 (0.96,2.49)	1.13 (0.85,3.27)	0.497
Tumor^#^	21	15 (51.7%)	6 (21.4%)	0.018
ESRD	8	3 (10.3%)	5 (17.9%)	0.47
60-day mortality	14	9 (31.0%)	5 (17.9%)	0.248

APACHE, acute physiology and chronic health evaluation; ANC, absolute neutrophil count; WBC, white blood count; ESRD, end-stage renal disease; * Direct lung injury is defined as ARDS risk factor of pneumonia or aspiration; ^**#**^Includes: solid metastatic, solid nonmetastatic, leukemia, lymphoma, and multiple myeloma.

### Identification of the differently expressed genes

**Figure 1:**
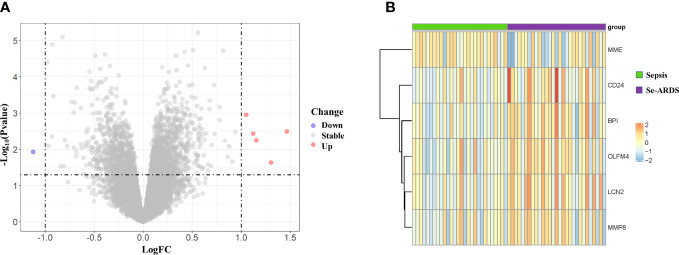
Identification of differently expressed genes (DEGs) associated with the onset of ARDS in septic patients. Six DEGs were identified between critically ill patients with sepsis alone and septic patients who developed ARDS, as visualized by volcano plot **(A)** as well as heatmap **(B)**.

**Figure 2:**
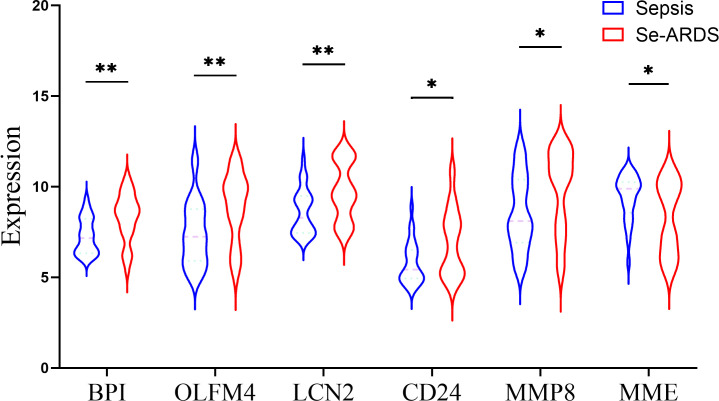
Comparison of DEGs expression between two groups. The expression level of DEGs were compared between patients with sepsis and patients with sepsis-induced ARDS (Se-ARDS), including BPI, OLFM4, LCN2, CD24, MMP8 and MME. Statistics are by Student’s *t* test. A two-tailed *p* value less than 0.05 is deemed as of statistical significance. *p < 0.05, **p < 0.01.

The DEGs were screened by ‘limma’ package with R software. The GSE66890 dataset contained six DEGs, including five upregulated genes: bactericidal permeability increasing protein (BPI), olfactomedin 4 (OLFM4), lipocalin 2 (LCN2), CD24, and matrix metallopeptidase 8 (MMP8), with one downregulated gene: membrane metalloendopeptidase (MME), as shown by the heatmap and volcano plot (**Figure 1**). The expression levels of these genes between the two groups were presented and compared in **Figure 2**. The septic patients who develop ARDS during their late course have significantly higher expression levels of BPI (7.27 ± 0.99 *vs*. 8.33 ± 1.35, *p*=0.001), OLFM4 (7.42 ± 1.79 *vs*. 8.88 ± 1.91, *p*=0.004), LCN2 (8.6 ± 1.25 *vs*.9.73 ± 1.59, *p*=0.005), CD24 (5.76 ± 1.16 *vs*. 6.92 ± 1.86, *p*=0.007), MMP8 (8.56 ± 2.05 *vs*. 9.86 ± 2.29, *p*=0.027) and lower levels of MME (9.42 ± 1.27 *vs*. 8.29 ± 1.99, *p*=0.014) than those not developed ARDS. However, due to distinct time points of sampling, there was no difference between septic patients and septic patients with ARDS (data from GSE10474 and GSE32707) (**Supplementary Figures 1**, **2**). Furthermore, we performed a gene network analysis based on 6 identified DEGs. As shown in **Supplementary Figure 3**, co-expressed and co-localized genes was predominantly enriched in GO terms, including leukocyte proliferation, defense response to bacterium and fugus, granulocyte migration as well as regulation of inflammation response.

### Identifying indicators for the onset of ARDS in septic patients

**Figure 3:**
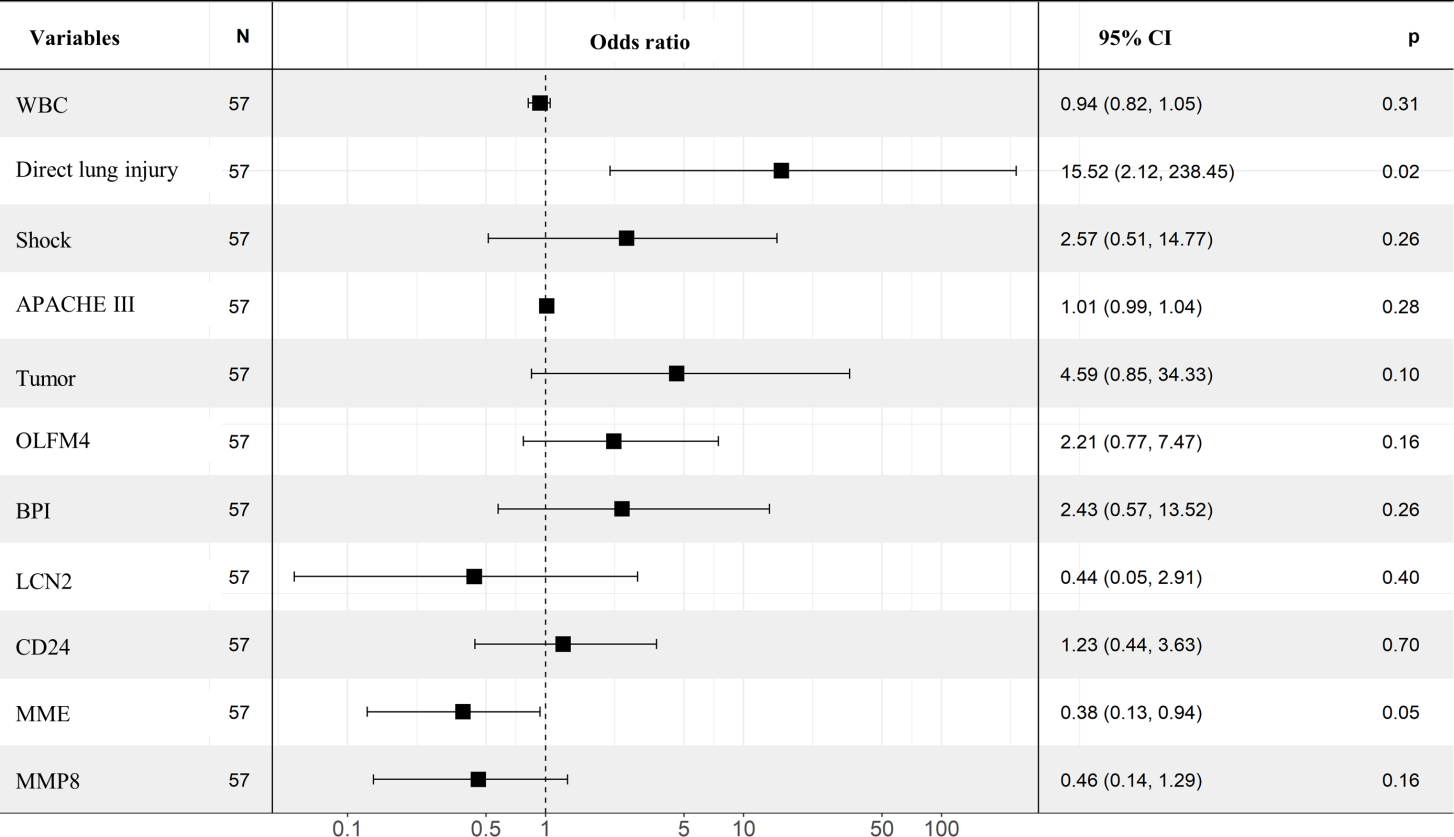
Multivariate analysis in identifying the predictors of ARDS. Forest plot showed the results of multivariate regression analysis after incorporating statistically significant factors in the univariate analysis, in which direct lung injury was identified as independent risk factor.

Thereafter, we applied univariate and multivariate regression analysis to select potential risk factors of ARDS occurrence in septic patients. As shown in **Table 2**, the univariate analysis results showed that the following variables were significant risk factors: direct lung injury (Odds ratio [OR]=3.029, 95% confidence interval [CI]=1.006-9.119, *p*=0.049), shock (OR=3.5, 95%CI =1.158-10.579, *p*=0.026), APACHE III scores (OR=1.025, 95%CI=1.008-1.042, *p*=0.003), WBC (OR=0.916, 95%CI =0.84-0.998, *p*=0.044), tumor (OR=3.929, 95%CI=1.232-12.531, *p*=0.021), BPI (OR=2.078, 95%CI=1.271-3.399, *p*=0.004), OLFM4 (OR=1.523, 95%CI=1.121-2.07, *p*=0.007), LCN2 (OR=1.715, 95%CI=1.15-2.557, *p*=0.008), CD24 (OR=1.648, 95%CI=1.115-2.435, *p*=0.012), MMP8 (OR=1.318, 95%CI=1.025-1.694, *p*=0.031), and MME (OR=0.667, 95%CI=0.475-0.936, *p*=0.019). However, multivariate regression analysis after adjusting for evaluated factors substantiated that only direct lung injury (OR=15.52, 95%CI=2.12-238.45, *p*=0.02) was significantly associated with the development of ARDS (**Figure 3**).

**Table 2:** Univariate analysis of the predictors for the development of ARDS.

Variables	OR	95%CI	p
Age	0.978	0.951-1.006	0.118
Gender (male *vs*. female)	0.923	0.324-2.629	0.881
Direct lung injury^*^	3.029	1.006-9.119	0.049
Shock	3.5	1.158-10.579	0.026
APACHE III	1.025	1.008-1.042	0.003
ANC	0.935	0.861-1.016	0.113
WBC	0.916	0.84-0.998	0.044
Creatinine	0.991	0.8-1.226	0.932
ESRD	0.531	0.114-2.469	0.419
Tumor^#^	3.929	1.232-12.531	0.021
BPI	2.078	1.271-3.399	0.004
OLFM4	1.523	1.121-2.07	0.007
LCN2	1.715	1.15-2.557	0.008
CD24	1.648	1.115-2.435	0.012
MMP8	1.318	1.025-1.694	0.031
MME	0.667	0.475-0.936	0.019

APACHE, Acute Physiology and Chronic Health Evaluation; ANC, absolute neutrophil count; WBC, white blood count; ESRD, end-stage renal disease; * Direct lung injury is defined as ARDS risk factor of pneumonia or aspiration; ^**#**^Includes: solid metastatic, solid nonmetastatic, leukemia, lymphoma, and multiple myeloma.

### Establishment of the nomogram for ARDS prediction in septic patients

**Figure 4:**
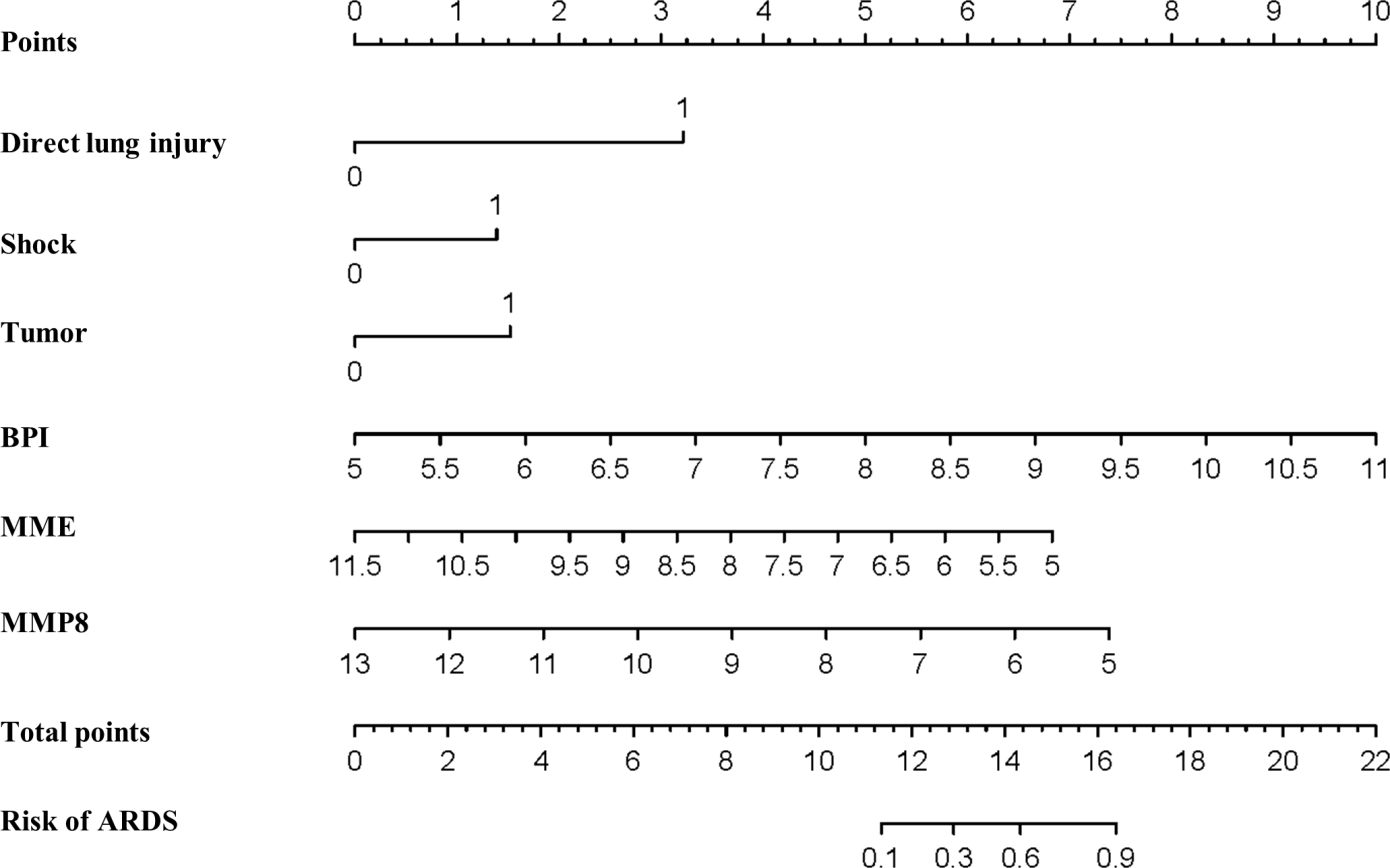
Establishment of the nomogram predicting ARDS in septic patients. Nomogram involving direct lung injury, shock, tumor, BPI, MME and MMP8 was visualized and applied for the prediction of ARDS among critically ill patients with sepsis.

**Figure 5:**
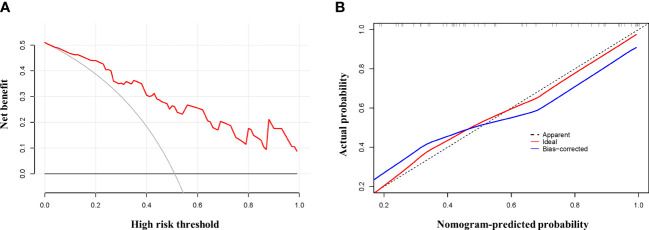
Testifying the net benefit and fitting of the nomogram. Calibration and decision curve analysis was used to determine the goodness of fit and net benefit of the nomogram, respectively. **(A)** Calibration plot of the nomogram. **(B)** DCA curve of the nomogram.

We fitted several multinomial logistic regression models of ARDS prediction using a backward stepwise selection method. The AIC of each model was presented in **Table 3**, in which the best-performing model corresponding to the minimum AIC was selected. According to the outcome prediction model, six valuable risk factors (direct lung injury, shock, tumor, BPI, MME and MMP8) were incorporated into the nomogram, which was used to predict the occurrence of ARDS in septic patients (**Figure 4**). The decision curve analysis and calibration curves were carried out to determine net benefit and predictive capacity of the nomogram. As shown in **Figure 5**, if the threshold probability is over 0.05, the septic patients who developed ARDS would benefit more from using this nomogram than the treating all or treating none scenarios. Besides, the calibration curve exhibited good consistency between predicted probability and observed probability, indicating optimal goodness of fit. Meanwhile, the c-index of the nomogram was 0.86, with a *p* value of 0.581 for Hosmer–Lemeshow goodness of fit test.

**Table 3:** Comparison of the performance across different models.

Models	AIC
Direct lung injury^*^ + Shock + APACHE III + Tumor^#^ + WBC+ OLFM4+ BPI+ LCN2+ CD24+ MME+ MMP8	70.85
Direct lung injury + Shock + APACHE III + Tumor + WBC+ OLFM4+ BPI+ LCN2+ MME+ MMP8	69.0
Direct lung injury + Shock + APACHE III + Tumor + WBC+ OLFM4+ BPI+ MME+ MMP8	67.58
Direct lung injury + Shock + APACHE III + Tumor + OLFM4+ BPI+ MME+ MMP8	66.41
Direct lung injury + Shock + APACHE III + Tumor + BPI+ MME+ MMP8	65.56
Direct lung injury + Shock + Tumor + BPI+ MME+ MMP8	64.83

APACHE, Acute Physiology and Chronic Health Evaluation; WBC, white blood count; * Direct lung injury is defined as ARDS risk factor of pneumonia or aspiration; ^**#**^ Includes: solid metastatic, solid nonmetastatic, leukemia, lymphoma, and multiple myeloma.

### Verifying the performance of the nomogram in predicting ARDS

**Figure 6:**
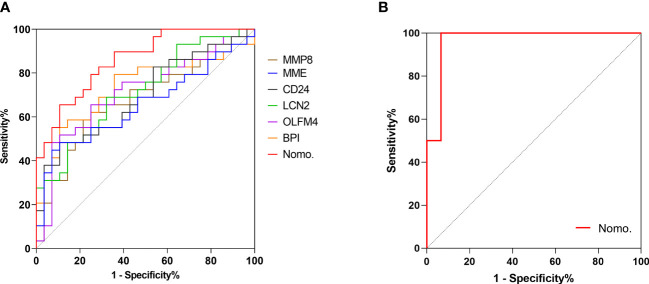
Validating the predictive value of the nomogram. Receiver operating characteristic (ROC) curve was adopted to verify the discriminatory capacity of the nomogram and sole use of each DEG in predicting ARDS incidence in septic patients. **(A)** ROC curve of the nomogram and each DEG in predicting ARDS occurrence among all septic patients. **(B)** ROC curve of the nomogram for ARDS prediction in septic patients without direct lung injury.

A point for each patient was calculated based on the nomogram and evaluated by ROC curve. As shown in **Figure 6A**, the area under the ROC (AUROC) for the prediction of ARDS occurrence in septic patients by the nomogram was 0.86 (95% CI=0.767-0.952). When the optimal cutoff point was set at 14.29, the corresponding specificity and sensitivity values were 89.29% and 65.52%, respectively. Furthermore, the discriminatory capacity of the nomogram was evidently superior than that of sole use of miRNA. Since direct lung injury represents one of common causes of ARDS, which may overlap with the contribution of sepsis, we therefore performed a sensitivity analysis evaluating the performance of this nomogram among septic patients without direct lung injury. As shown in **Figure 6B**, the AUROC for the prediction of ARDS in septic patients without direct lung injury was 0.967 (95% CI=0.896-1.0). To validate the robustness of our model, we repeated the analysis in predicting the 60-day mortality for all septic patients, in which the nomogram had an AUROC of 0.796 (95% CI = 0.66-0.932), indicating an acceptable performance (**Supplementary Figure 4**).

## Discussion

In the present study, by analyzing the microarray data of whole blood samples collected from critically ill septic patients with and without ARDS development, we identified several genes that were differentially expressed upon ICU admission. As the samples were collected before the development of ARDS in this cohort, we identified that the septic patients who would develop ARDS lately had increased expression of BPI, OLFM4, LCN2, CD24, MMP8 and decreased expression of MME at early time. Moreover, we combined significant clinical indicators (direct lung injury, shock and tumor) and genetic biomarkers (BPI, MME and MMP8) to generate a predictive model of ARDS in patients with sepsis, which showed good performance in terms of discriminatory capacity and goodness of fit.

Based on the public data GSE32707 dataset from GEO, Ming et al. identified SIGLEC9, TSPO, CKS1B, and PTTG3P as top ranking DEGs between patients with sole sepsis and those with Se-ARDS (9). Chen et al. screened out twelve DEGs by comparing chip data containing samples of acute lung injury with sepsis and samples of sepsis alone from GSE10474 dataset. Using the “limma” package for differential expression analysis, we identified six DEGs between patients with sepsis and those developing Se-ARDS. Different from previous studies, the blood samples in the present study were collected in septic patients before the development of ARDS, which partially explained why we failed to validate the expressions of these six DEGs using the GSE32707 and GSE10474 datasets. This divergency implied that these DEGs in our findings might critically involve in progression process to ARDS in sepsis.

In the original study, Kangelaris et al. have demonstrated that these DEGs were neutrophil-related genes and mediators of innate immune response, in consistent with our findings in gene network analysis (12). The role of these DEGs in sepsis or ARDS have been investigated and described previously. BPI is a cationic protein isolated from human neutrophils that binds lipopolysaccharide (LPS), thereby neutralizing many of the effects of LPS and ameliorating endotoxin effects (13, 14). Although autoantibodies targeting both the N-terminal domain and C-terminal domain of BPI could inhibit the functional activity of BPI, targeting the hinge region of BPI could enhance bacterial clearance (15). In a previous study, recombinant BPI treatment was shown to inhibit endotoxin-induced activation of circulating neutrophil and diminish the production of inflammatory cytokines, thereby alleviating acute lung injury (16). Correspondingly, it suggested a therapeutic potential of targeting BPI for the treatment of ARDS patients. OLFM4, an important regulator of inflammatory and immune responses, is highly expressed in various inflammatory diseases (17). In an LPS-challenge rat model, blockage of OLFM4 expression was confirmed to mitigate lung tissue damage and hyperinflammatory response (18). Additionally, Higher percentages of OLFM4^+^ neutrophils were shown to be associated with worsening clinical outcomes in patients with sepsis, blunt traumatic injuries, and ARDS (19, 20). LCN2, also known as neutrophil gelatinase-associated lipocalin (NGAL), has been identified as a key player in antimicrobial process, oxidative stress and inflammation (21, 22). Studies have revealed that LCN2 inhibition or silencing could exert protective effect on LPS-induced ARDS model *via* inhibition of ferroptosis-related inflammation and oxidative stress (23). Meanwhile, LCN2 was significantly elevated and positively correlated with disease severity in patients with influenza or COVID-19 (24). Interestingly, LCN2 expression is markedly upregulated in septic ALI mice compared with those without ALI, which is consistence with our findings (25). These results implied that LCN2 might play a pivotal role in the pathogenesis of ARDS. The CD24 molecule represents the neutrophil ligand of P-selectin, which is deemed as a regulator of heterotypic neutrophil-platelet and platelet-endothelial cell interactions. The expression of CD24 may serve as an indicator of vascular inflammation in ARDS (26, 27). MMP8, belongs to the member of the MMP family, is expressed predominantly by neutrophils. Quintero et al. showed that MMP8 regulated the accumulation of neutrophils and macrophages in the lung tissue during LPS-induced lung injury (28). Furthermore, inhibiting MMP8 activity or reduction in expression level of MMP8 in the lungs could restrain lung fibrotic responses to injury (29). In pediatric patients with ARDS, MMP8 expression levels were elevated, in association with deteriorative outcomes (30–32). MME, also known as neutral endopeptidase (NEP), is an enzyme that cleaves inflammatory bioactive peptides. Emerging evidence revealed that NEP played an protective role in ARDS, pharmacological inhibition of which exacerbated lung vascular leakage in mice with smoke inhalation injury (33), also evidenced by exaggerated acute pancreatitis-associated lung injury in NEP-deficient mice (34). Similar to our results, Hashimoto et al. found a substantial decrease in the peripheral activity of NEP. However, they found its intra-alveolar activity was significantly increased (35).

Although there were numerous studies exploring the biomarkers for prediction of the onset of ARDS, none has been universally accepted. There is an agreement that the combination of indicators results in better performance than the sole use of biomarkers. Fremont et al. reported a panel of seven cytokines, including receptor for advanced glycation end-products (RAGE), procollagen peptide III (PCPIII), brain natriuretic peptide (BNP), angiopoietin-2 (Ang-2), interleukin-10 (IL-10), tumor necrosis factor alpha (TNF-α) and interleukin-8 (IL-8), with high diagnostic accuracy for ARDS occurrence (4). Likewise, Villar et al. constructed a predictive model by combining RAGE, C-X-C Motif Chemokine Ligand 16 (CXCL16), Ang-2 and PaO_2_/FiO_2_, facilitating ARDS prediction among septic patients (36). In considering of the cost-effectiveness and multicollinearity of the incorporated biomarkers reflecting similar pathophysiology, Fu et al. recently found that the diagnostic value of ARDS improved when combining C-reactive protein (CRP), Ang-2, clara cell secretory protein (CC16), high mobility group protein 1 (HMGB1) and PaO_2_/FiO_2_ (37). However, exclusively inclusion of known protein biomarkers may inevitably lead to the limitations of the advances in biomarkers research and clinical application progress on ARDS. To comprehensively enhance predictive accuracy and develop a multi-parametric prediction, the current study applied bioinformatics analysis by combining demographics and clinicopathological features with transcriptomic data. The microarray data and bioinformatics analysis help uncovering the in-depth pathogenesis and advancing the discovery of new biomarkers. Unexpectedly, according to multivariate regression analysis, only direct lung injury was demonstrated to be an independent risk factor for the onset of ARDS. Since multicollinearity across distinct indicators may exist, holistic incorporation of parameters that are of statistically significant in univariate regression analysis into a single model could inevitably eliminate their potential in predicting endpoints. To avoiding underfitting and overfitting of the model, we therefore constructed and screened multinomial logistic regression models using backward stepwise selection method in line with the AIC principle, followed by visualization using a nomogram (38). Consequently, our results showed that the nomogram based on direct lung injury, shock, tumor, BPI, MME and MMP8, had incremental predictive value compared to that of the single indicators. The nomogram performs well in recognizing the complication of ARDS in septic patients, with a c-index of 0.860, indicating a satisfactory discriminatory capacity. The Hosmer-Lemeshow goodness of fit test showed a good consistency (*p*=0.581). Moreover, after excluding septic patients with direct lung injury, the nomogram also showed an acceptable performance in predicting ARDS.

We acknowledged several limitations when interpretating key findings in the present study. Firstly, since this is a secondary analysis based on previously reported dataset, we have no access to the detailed patient records and may not account for other factors associated with ARDS occurrence. Secondly, the sample size is relatively modest, which may increase the opportunity underlying the influence of confounding factors. Thirdly, the genes were detected only in circulating leukocytes but not in other cells known to be involved in the pathogenesis of lung injury. Future studies assessing single cell transcriptomics of other cells such as endothelial and epithelial cells are clearly warranted. Most importantly, the findings of this study require further validation in an independent and external datasets. Despite these limitations, the striking capacity of this nomogram in predicting the development of ARDS among septic patients highlights the superiority of generating a model combining transcriptional biomarkers and clinical parameters.

In summary, we identified six genes that were differentially expressed upon ICU admission between septic patients with or without the development of ARDS. A nomogram combining transcriptional biomarkers and clinical parameters was established and showed a favorable performance in predicting the onset of ARDS in patients with sepsis.

## Data availability statement

The datasets presented in this study can be found in online repositories. The names of the repository/repositories and accession number(s) can be found in the article/**Supplementary Material**.

## Author contributions

G-SW conceived this analysis. ZS, Q-MM, and PL were responsible for the data interpretation. G-SW and R-QY co-wrote the paper. G-SW, R-QY and C-RW undertook the statistical analyses and analyzed the data. YD, L-YZ, and WL refined the methodologies. FZ and YS supervised the study and provided critical revision of the manuscript. All authors contributed to the article and approved the submitted version.

## Supplementary material

Supplementary Figure 1 Comparing the expression levels of BPI, OLFM4, LCN2, CD24, MMP8, and MME between septic patients and septic patients with ARDS in GSE10474.

Supplementary Figure 2 Analysis of the expression levels of BPI, OLFM4, LCN2, CD24, MMP8, and MME in GSE32707.

Supplementary Figure 3 Gene network analysis based on DEGs. Gene network was constructed based on 6 identified DEGs, including BPI, OLFM4, LCN2, CD24, MMP8, and MME, showing co-expressed and co-localized genes, with functional annotation in line with Gene Ontology (GO) category.

Supplementary Figure 4 ROC curve showing the discriminatory capacity of the nomogram in predicting 60-day mortality for septic patients.
