# Supplementary figures and images for: Combination of transcriptional biomarkers and clinical parameters for early prediction of sepsis indued acute respiratory distress syndrome
Source: Front Immunol. 2023 Jan 4;13:1084568. doi: 10.3389/fimmu.2022.1084568 (PMC9846102; doi:10.3389/fimmu.2022.1084568)

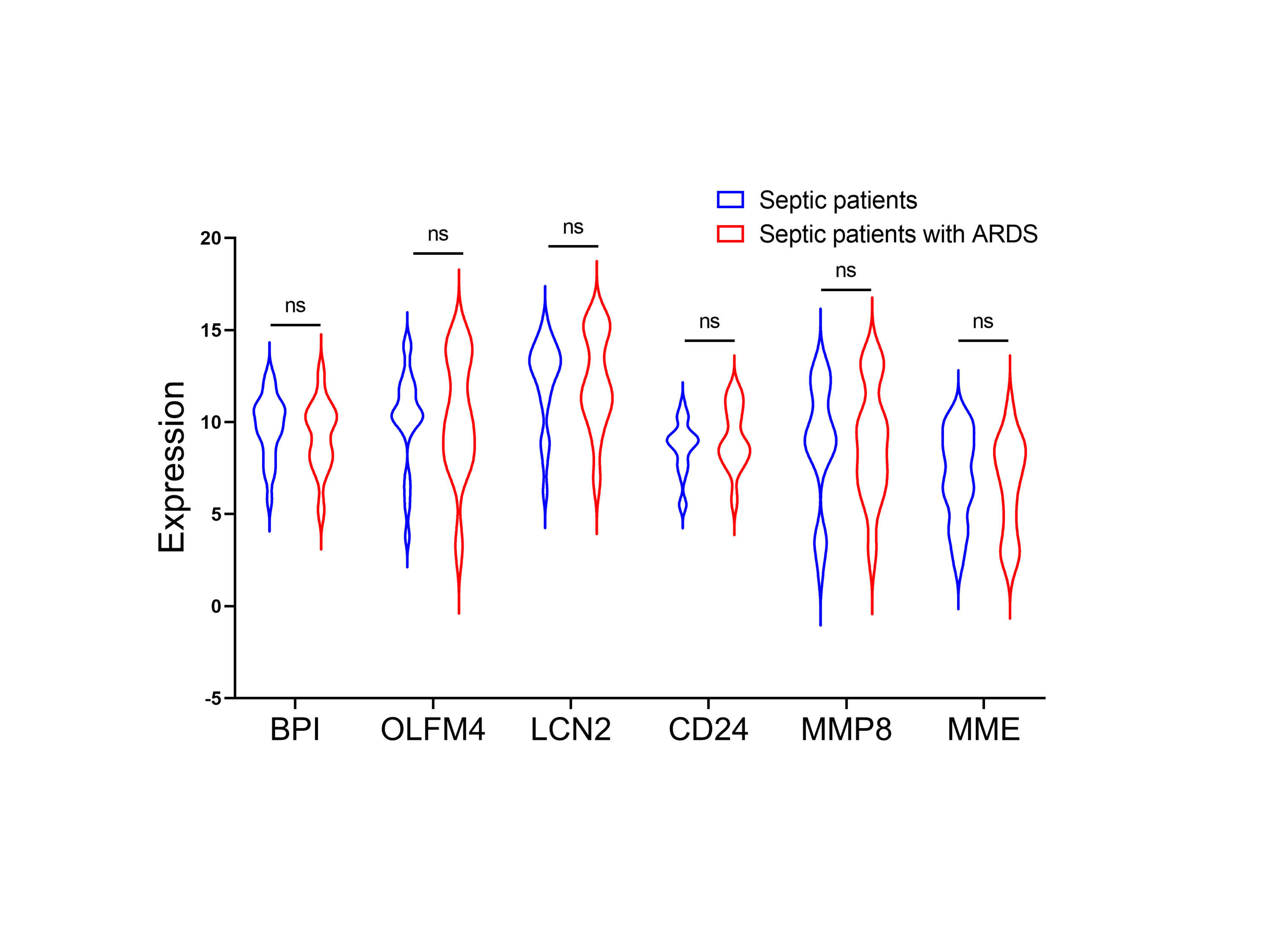

Supplement: Supplementary Figure 1 — Comparing the expression levels of BPI, OLFM4, LCN2, CD24, MMP8, and MME between septic patients and septic patients with ARDS in GSE10474. [file Image_1.tif]

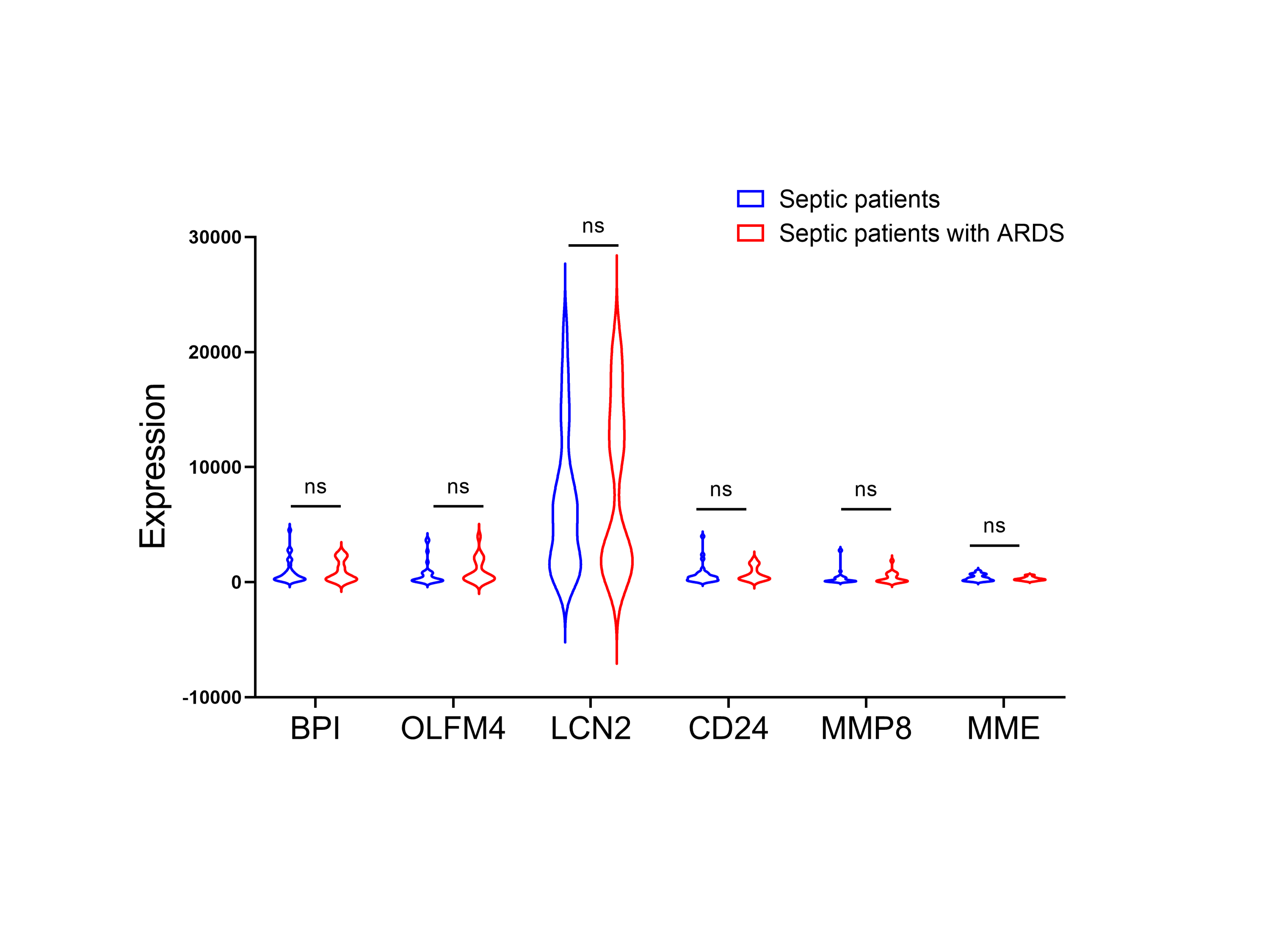

Supplement: Supplementary Figure 2 — Analysis of the expression levels of BPI, OLFM4, LCN2, CD24, MMP8, and MME in GSE32707. [file Image_2.tif]

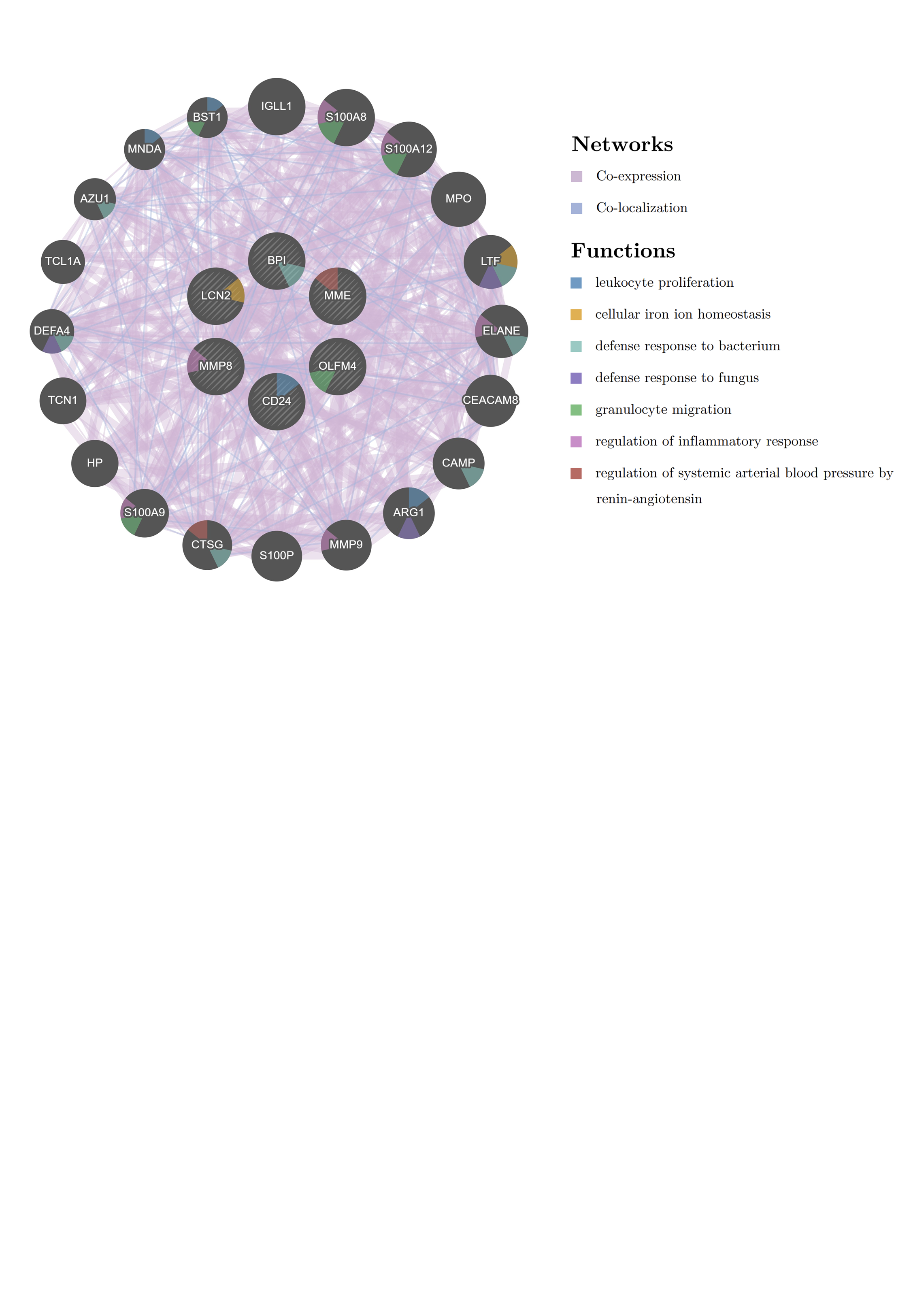

Supplement: Supplementary Figure 3 — Gene network analysis based on DEGs. Gene network was constructed based on 6 identified DEGs, including BPI, OLFM4, LCN2, CD24, MMP8, and MME, showing co-expressed and co-localized genes, with functional annotation in line with Gene Ontology (GO) category. [file Image_3.tif]

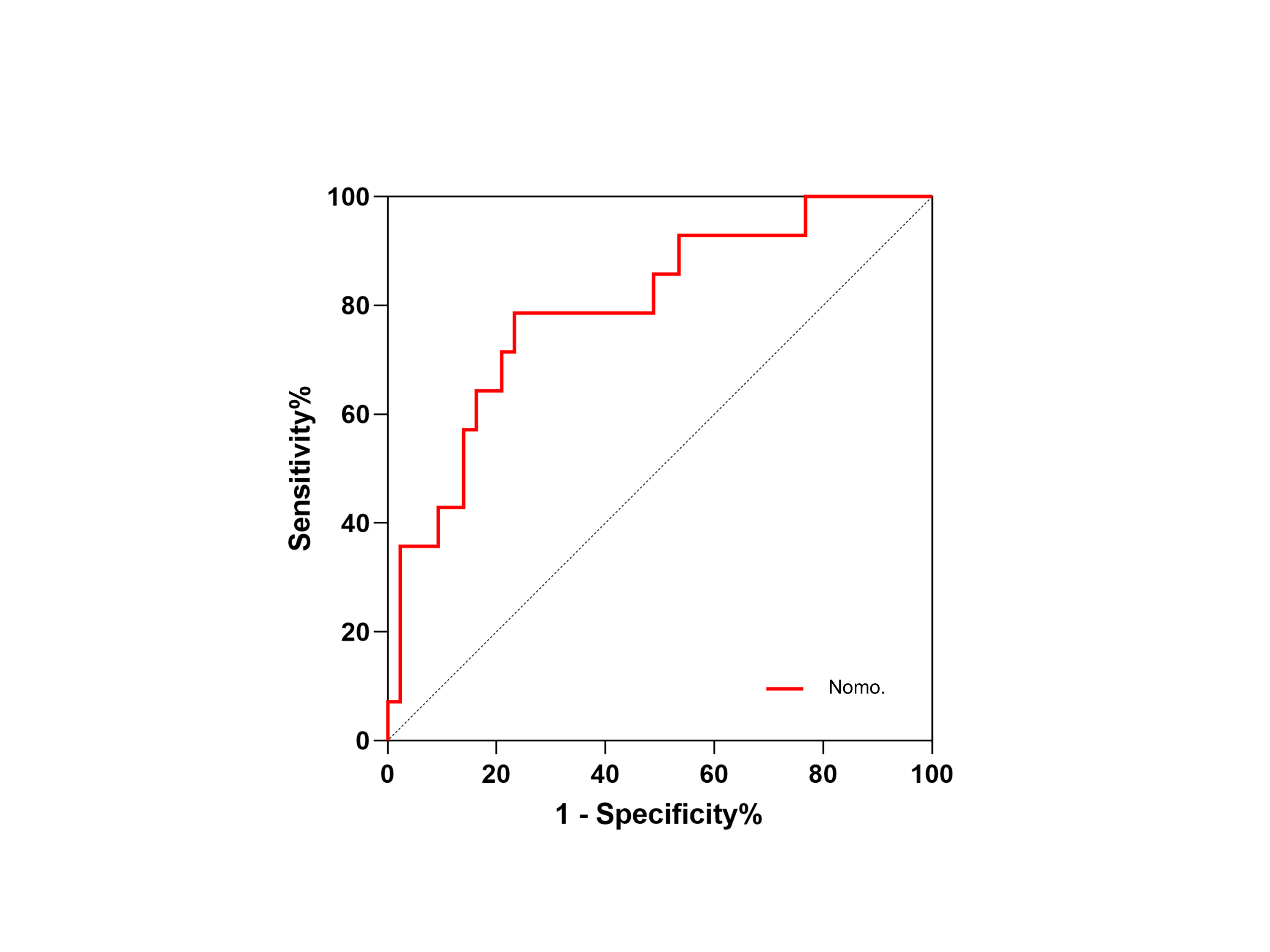

Supplement: Supplementary Figure 4 — ROC curve showing the discriminatory capacity of the nomogram in predicting 60-day mortality for septic patients. [file Image_4.tif]
